# Neutrophil extracellular traps predict postoperative pulmonary complications in paediatric patients undergoing parental liver transplantation

**DOI:** 10.1186/s12876-023-02744-0

**Published:** 2023-07-13

**Authors:** Yaling Liu, Huigang Shu, Ping Wan, Xiaodong Wang, Hong Xie

**Affiliations:** 1 Department of Anesthesiology, The Second Affiliated Hospital of Soochow University, 1055 Sanxiang Road Suzhou, Jiangsu, China; 2 Department of Anesthesiology, Renji Hospital, Shanghai Jiaotong University School of Medicine, 160 Pujian Road, Shanghai, 200127 China; 3 Department of Liver Surgery, Renji Hospital, Shanghai Jiaotong University School of Medicine, Shanghai, China; 4 Department of Cardiology, Shanghai East Hospital, Tongji University School of Medicine, 150 Jimo Road, Shanghai, China

**Keywords:** Parental liver transplantation, Postoperative pulmonary complication, Paediatric

## Abstract

**Background:**

Parental liver transplantation (PLT) improves long-term survival rates in paediatric hepatic failure patients; however, the mechanism of PLT-induced postoperative pulmonary complications (PPCs) is unclear.

**Methods:**

A total of 133 paediatric patients undergoing PLT were included. Serum levels of NET components, including circulating free DNA (cfDNA), DNA-histone complex, and myeloperoxidase (MPO)-DNA complex, were detected. The occurrence of PPCs post-PLT, prolonged intensive care unit (ICU) stay and death within one year were recorded as the primary and secondary outcomes.

**Results:**

The overall rate of PPCs in the hospital was 47.4%. High levels of serum cfDNA, DNA-histone complexes and MPO-DNA complexes were associated with an increased risk of PPCs (for cfDNA, OR 2.24; for DNA-histone complex, OR 1.64; and for MPO-DNA, OR 1.94), prolonged ICU stay (OR 1.98, 4.26 and 3.69, respectively), and death within one year (OR 1.53, 2.65 and 1.85, respectively). The area under the curve of NET components for the prediction of PPCs was 0.843 for cfDNA, 0.813 for DNA-histone complexes, and 0.906 for MPO-DNA complexes. During the one-year follow-up, the death rate was higher in patients with PPCs than in patients without PPCs (14.3% vs. 2.9%, *P* = 0.001).

**Conclusions:**

High serum levels of NET components are associated with an increased incidence of PPCs and death within one year in paediatric patients undergoing PLT. Serum levels of NET components serve as a biomarker for post-PLT PPCs and a prognostic indicator.

## Introduction

The surgical technique of paediatric parental liver transplantation (PLT) in appropriate patients has achieved dramatic advancements over the last several decades. PLT significantly increases long-term survival rates, with more than 85% in most large paediatric transplantation centres [1]. However, post-PLT patients are at high risk of postoperative pulmonary complications (PPCs), including pleural effusion, pulmonary oedema, atelectasis, pneumonia, and especially acute respiratory distress syndrome (ARDS) [2, 3]. In adult liver transplantations, contributing factors to PPCs include systemic inflammatory response, haemodynamic impairment, reperfusion syndrome, thrombosis and early graft dysfunction [4, 5]. However, the mechanisms associated with PPCs after paediatric PLT have not been clearly identified. Since PPCs are associated with postsurgical death [6, 7], it is important to find biomarkers that could predict the occurrence of PPCs and PLT-related death.

Polymorphonuclear neutrophils are important players in the response to infection, trauma and other stimuli. Neutrophil extracellular traps (NETs) have recently been reported to participate in the process of lung injury [8], pneumonia [9] and pulmonary oedema [10]. NETs are produced by neutrophils through the release of their intracellular contents, such as DNA, histones [11] and myeloperoxidase (MPO) [12]. These structures form a large net-like scaffold named NETs in which pathogens become trapped. If these NETs become overwhelming, tissue damage will follow. Since the lung is the location where marginating neutrophils reside, it is particularly susceptible to NET-related injury [13]. NETs play a complicated role in various lung diseases. For example, the NET component MPO-DNA complexes in the alveoli is associated with ventilator-associated pneumonia (VAP) in patients on mechanical ventilation [9]. Interestingly, circulating histones could directly mediate trauma-associated lung injury [8]. In enterovirus 71-induced pulmonary oedema in hand, foot, and mouth disease, the NET components citrullinated histone 3 and MPO have both harmful and beneficial effects on pulmonary oedema [10].

Both human and animal studies have suggested that pulmonary thrombosis and platelet aggregation are involved in ARDS after sepsis [14]. Preclinical studies have shown that anticoagulation may improve the outcome of ARDS [15]. However, the underlying mechanisms remain largely unknown. Activation of neutrophils by microbial or inflammatory stimuli during lung injury results in the release of NETs [16]. Research has shown that NETs promote thrombosis through platelet-dependent and platelet-independent mechanisms. For example, NETs have been suggested to play an active role in the development of thrombotic diseases, such as deep vein thrombosis, myocardial infarction, and stroke, by activating tissue factor or impairing tissue plasminogen activator-induced thrombolysis [17–19]. Because liver transplantation is related to hepatocyte death and intrahepatic neutrophil accumulation, NETs may affect the haemostatic balance post liver transplantation.

Given the increased mortality related to post-PLT PPCs and the pathobiological link between NETs and PPCs, we conducted the present prospective study to explore the potential value of NET components as biomarkers for post-PLT PPCs. Serum concentrations of NET components before and 24 h after PLT were measured, and their association with post-PLT PPCs was analysed.

## Materials and methods

### Study design and subjects

This prospective study was conducted at Renji Hospital, School of Medicine, Shanghai Jiaotong University from July 1, 2019, to October 10, 2020. The study protocol was approved by the Renji Hospital Ethics Committee and adhered to the principles of the Declaration of Helsinki. Written informed consent was obtained from the patients’ guardians on the day before surgery. Inclusion criteria were as follows: 0–1 year old, needing PLT as a life-saving surgical intervention, and no contraindications to surgery. Exclusion criteria included any of the following: respiratory tract infection in the 2 weeks before the operation, abnormalities on preoperative chest X-ray, congenital respiratory diseases, or refusal to sign informed consent. Serum samples were collected twice 24 h apart.

### Serum sample collection

Serum samples were collected before and 24 h after PLT. In brief, blood was drawn via venipuncture or central venous catheter into a 3.2% sodium citrate tube. The blood samples were centrifuged at 1,500 × g for 10 min at room temperature, and supernatants were collected and transferred into new collection tubes. Serum samples were stored at –80 °C until they were used for analysis.

### Neutrophil isolation and preparation

Neutrophils from peripheral blood were isolated using a neutrophil isolation kit by following a protocol provided by the manufacturer (P9040, Solarbio). Red blood cells were lysed with red blood lysis buffer (8.3 g NH_4_Cl, 1.0 g KHCO_3_, and 0.5 M EDTA). RPMI 1640 plus 10% FBS was used as the culture medium for all reactions.

### Measurement of NET components

Serum levels of NET components, including cfDNA, DNA-histone complex, and MPO-DNA complex, were measured as follows.

#### Measurement of serum cfDNA

The concentration of cfDNA was measured using the Quant-iT PicoGreen double-stranded DNA assay kit (Sarstedt, Nümbrecht, Germany). Briefly, 3 μL of patient serum was added to each microwell containing 100 μL of Tris–EDTA (TE) buffer (10 mM Tris–HCl, 1 mM EDTA, pH 7.5). Next, 100 μL of PicoGreen solution (diluted 1:200 in TE buffer) was added to the wells. The reaction mixture was incubated in darkness for 5 min, and the fluorescence intensity was measured at 480 nm excitation and 520 nm emission using a Victor (PerkinElmer, Groningen, the Netherlands) photometer.

#### Measurement of serum DNA-histone complexes

Serum DNA-histone complex levels were measured using a commercially available sandwich enzyme-linked immunosorbent assay (ELISA) (Cell Death Detection ELISAplus, Sigma‒Aldrich, St. Louis, MO, USA) according to the manufacturer’s instructions. Briefly, 20 μL of serum was diluted 1:4 in the immunoreagent that was prepared by mixing 5% volume of peroxidase-conjugated anti-DNA antibody, 5% volume of biotin-conjugated anti-histone antibody, and 90% volume of incubation buffer. Diluted serum was then added to streptavidin-coated microtiter plates, where the streptavidin captured the biotin. After 3 h of incubation and sufficient washing with PBS, the peroxidase activity of the retained immunocomplexes was measured by incubation with ABTS (2,2′-azino-di[3-ethylbenzthiazoline- sulfonate]), the substrate of peroxidase, yielding a green end-product. The optical density (OD) was read in a spectrophotometer at 405 nm [19].

#### Measurement of serum MPO-DNA complexes

Serum MPO-DNA complex levels were measured by “sandwich” ELISA as previously described [20]. In brief, the wells of ELISA microplates (Nunc MaxiSorp Prod. #439,454, or Corning Prod. #3590) were coated with an MPO-specific monoclonal antibody (Abcam, ab45977, Cambridge, MA, USA) at 1 μg/ml in carbonate buffer (100 μl/well) and immediately sealed and incubated at 4 °C overnight. After blocking and sufficient washing, 100 μl of standard or serum samples was added to each well in triplicate. After incubation at room temperature for at least 2 h, 100 μl of peroxidase-conjugated anti-DNA monoclonal antibody (Roche Diagnostics, Indianapolis, IN, USA) was added at a concentration of 0.5 μg/ml in diluent (0.05% Tween-20, 0.1% BSA in PBS). After 2 h of incubation at room temperature, the wells were washed, and 100 μl of ABTS substrate solution was added and incubated at room temperature for colour development. The OD 405 nm was read on a spectrophotometer.

### Immunostaining and confocal microscopy

Neutrophils (4 × 10^5^) were seeded on a sterile round glass coverslip that was placed in a 24-well cell culture plate. Phorbol myristate acetate (PMA; 100 nM) (Sigma, USA) was added as a positive control to stimulate NET formation. After 4 h of incubation, the glass coverslips with the attached cells were carefully removed from a 24-well culture plate and fixed with ice-cold 4% PFA. Then, samples were blocked and stained with a mouse monoclonal antibody against histone H3 (1:400, 14269S, Cell Signalling) and with a rabbit polyclonal antibody against myeloperoxidase (1:200, ab45977, Abcam). The samples were washed and further stained with an Alexa Fluor® 488 goat anti-rabbit antibody (1:1000, Life Technologies, USA) and an Alexa Fluor® 647 goat anti-mouse antibody (1:1000, Life Technologies, USA). Nuclei in the samples were stained with 4′6-diamidino-2-phenylindole. Images were captured by an Olympus ((BXFM, Tokyo, Japan) confocal fluorescence microscope using the appropriate lenses and filters.

### Clinical endpoints

The primary endpoint was the incidence of PPCs at 7 days post-operation, defined as the occurrence of pleural effusion, pulmonary oedema, atelectasis, pneumonia and/or ARDS. Briefly, PPCs were diagnosed as follows [7, 21]: 1) Pleural effusion – chest X-ray demonstrating blunting of the costophrenic angle, loss of the sharp silhouette of the ipsilateral hemidiaphragm in the upright position, with or without displacement of adjacent anatomical structures; 2) Pulmonary oedema – pulmonary vascular texture thickening vague (“Butterfly wing sign”), cuff sign of bronchus; 3) Atelectasis – lung opacification and compensatory overinflation in the adjacent nonatelectatic lung; 4) Pneumonia – chest radiograph showing changes or new patchy infiltrates, lobar or segmental consolidation, ground-glass opacities, or interstitial changes, with or without pleural effusion and met at least one of the following criteria: fever or leukocyte count at least 12*10^9^/L [22]; and 5) ARDS was defined according to the criteria of bilateral opacities not fully explained by effusions, lobar/lung collapse, or nodules, excluding cardiac failure or fluid overload-pulmonary oedema via transthoracic echocardiography [23]. The secondary endpoints were prolonged stay in the ICU (more than 3 days) [24] and all-cause death within 1 year post-surgery.

### Statistical analyses

Statistical analyses were performed using SPSS statistics, version 17.0 (IBM Inc., Chicago, IL) and GraphPad Prism 5.0 (GraphPad Software, Inc., San Diego, CA). Continuous variables with a normal distribution are presented as the mean ± SD and were analysed with unpaired Student’s t test (2 groups) or one-way analysis of variance (ANOVA, more than 2 groups). Continuous variables with skewed distributions are presented as medians and interquartile ranges and were analysed with the Wilcoxon signed-rank test. Categorical variables are presented as percentages and were analysed with the χ^2^ test. Correlations between NET components and clinical endpoints were made using logistic regression analysis. In brief, for cfDNA, 1–5 represented 0–999 ng/mL, 1000–1999 ng/mL, 2000–2999 ng/mL, 3000–3999 ng/mL and > 4000 ng/mL, respectively. For DNA-histone complexes and MPO-DNA complexes, 1–5 represented 0–0.99 OD, 1–1.99 OD, 2–2.99 OD, 3–3.99 OD and 4–4.99 OD, respectively. Kaplan‒Meier survival curves during the first year after PLT surgery were generated. A P value < 0.05 was considered statistically significant.

## Results

### Study population

**Fig. 1:**
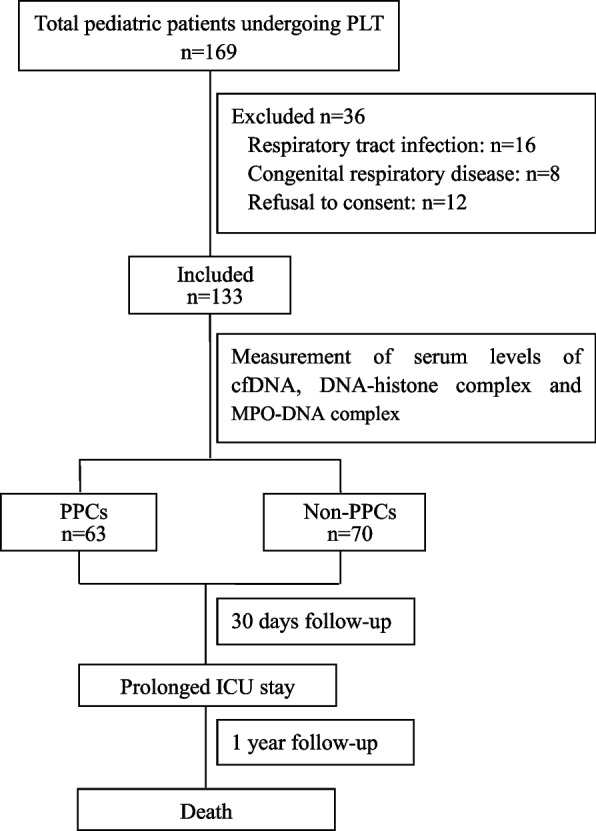
Flow chart of the study design of the present study

From July 1, 2019, to October 10, 2020, 169 paediatric patients were scheduled for PLT surgery, and 36 patients were excluded. Eventually, a total of 133 patients undergoing liver surgery were included in our analysis, and the patients were followed-up for one year after surgery. Six-three patients (47.4%) were diagnosed with PPCs, and seventy patients had no PPCs. No patients were lost to follow-up (Fig. 1). Among them, 45.1% were male. The average age was 0.8 ± 0.3 years old, and the weight was 8.1 ± 2.4 kg. Indications for PLT included extrahepatic biliary atresia, progressive familial intrahepatic cholestasis, acute liver failure, Wilson’s disease, α-1 antitrypsin deficiency, tyrosinaemia, and primary liver tumours. As shown in Table 1, the baseline demographic characteristics and underlying liver diseases were comparable between PPC patients and non-PPC patients.

**Table 1:** The category for liver transplantation, PPCs and NETs concentration

Patient characteristics and pathological features	Total N	Post-operative pulmonary complications		*P* value
		Yes	No	
Total cases	133	63	70	0.027
Age (years)	0.8 ± 0.3	0.9 ± 0.5	0.7 ± 0.4	0.302
Weight (Kg)	8.1 ± 2.4	7.3 ± 2.3	8.9 ± 2.5	0.518
Male sex	60(45.1%)	29(46.0%)	31(44.3%)	0.883
Transfusion volume (ml)	145 ± 17	198 ± 42	134 ± 28	0.580
Underlying liver diseases				
Extra-hepatic biliary atresia	50(38%)	24(38%)	26(38%)	0.417
Progressive familial intrahepatic cholestasis	33(25%)	17(26%)	16(23%	0.109
Acute liver failure	20(15%)	8(13%)	12(16%)	0.620
Wilson's disease	17(13%)	8(13%)	9(12%)	0.824
Alpha-1 antitrypsin deficiency	7(5%)	3(5%)	4(6%)	0.988
Tyrosinemia	3(2%)	2(3%)	1(2%)	0.476
Primary liver tumors	3(2%)	1(2%)	2(3%)	0.583
PPCs				
Pleural effusion	20(15%)	20(32%)	0	< 0.001
Pulmonary edema	9(6%)	9(14%)	0	< 0.001
Atelectasis	9(6%)	9(14%)	0	< 0.001
Pneumonia	10(8%)	10(16%)	0	< 0.001
ARDS	15(11%)	15(24%)	0	< 0.001
Operation time (min)	262.1 ± 46.8	290.9 ± 63.6	272.9 ± 56.3	0.703
ICU duration (d)	5	7	3	0.03
1 year death	11(8.3%)	9(14.3%)	2(2.9%)	0.001

*AA* Arachidonic acid

*ADP* Adenosine diphosphate

*ARDS* Acute respiratory distress syndrome

*cfDNA* Circulating free DNA

*ICU* Intensive care unit

*MPO* Myeloperoxidase

*NETs* Neutrophil extracellular traps

*PPCs* Postoperative pulmonary complications

### Relative levels of NET components in PPC and non-PPC patients

**Fig. 2:**
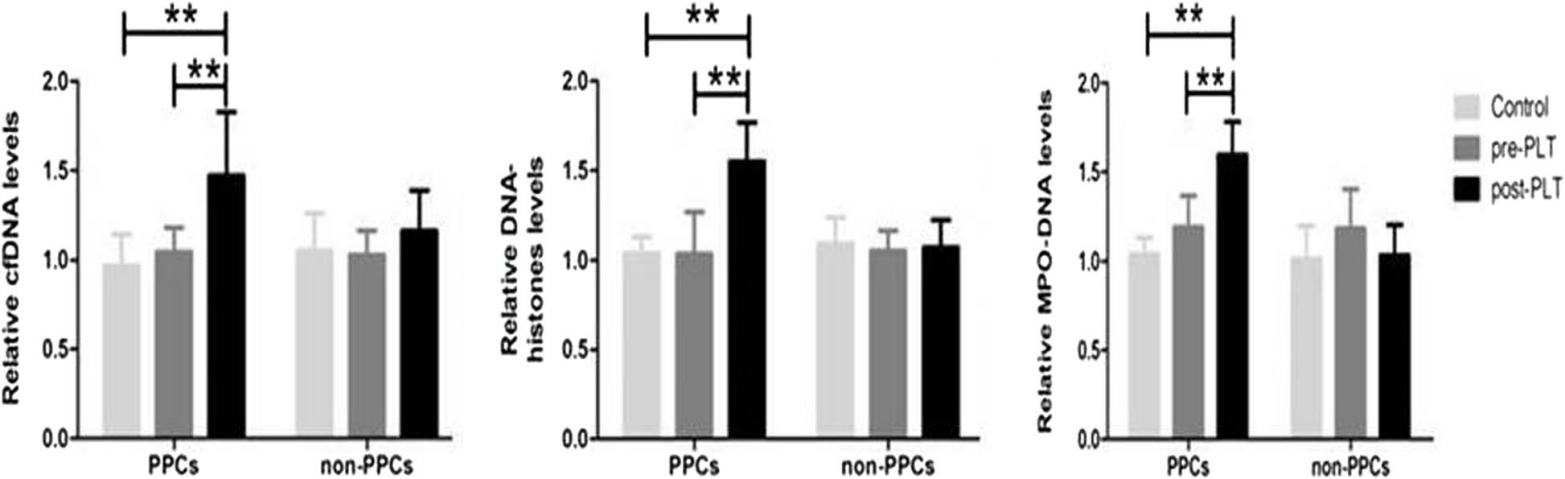
Serum levels of NET components in patients with or without PPCs. Serum levels of the NET components cfDNA, DNA-histone complexes, and MPO-DNA complexes in healthy controls and PLT patients with or without PPCs. The levels both pre-PLT and 24 h post-PLT were measured. Data are the mean ± SD, expressed relative to healthy controls. ** *p* < 0.01. One-way ANOVA with Bonferroni post hoc analysis. NET, neutrophil extracellular trap; cfDNA, circulating free DNA; MPO, myeloperoxidase; PLT, parental liver transplantation; PPCs: postoperative pulmonary complications.

The concentrations of serum NET components, including cfDNA, DNA-histone complexes and MPO-DNA complexes, were detected in healthy control children and PLT patients both preoperatively and at 24 h postoperatively. As shown in Fig. 2, for all three NET components, no difference was noted between healthy controls and the preoperative baseline levels in patients. Interestingly, in patients with PPCs, the concentrations of all three NET components increased dramatically at 24 h after surgery in the PPC group compared with baseline before PLT (*p* < 0.01), which was not observed in non-PPC patients. This finding suggests that the serum levels of post-PLT NET components may serve as a biomarker for the development of PPCs. To test this hypothesis, we then performed regression analysis of post-PLT NET components with the occurrence of PPCs.

### Regression analysis of post-PLT NET component levels with the primary endpoint: PPCs

Using univariate logistic regression analysis, we found that patients with high serum levels of cfDNA (OR 2.36, 95% CI 1.82–4.68, *P* < 0.001), DNA-histone complexes (OR 1.65, 95% CI 1.18–2.30, *P* = 0.003) and MPO-DNA complexes (OR 2.91, 95% CI, 1.58–3.52, *P* = 0.004) were at higher risk of developing PPCs at 7 days post-surgery (Table 2). In the multivariate logistic regression model, patients with high serum levels of cfDNA (OR 2.24; 95% CI 1.37–3.65, *P* = 0.004), DNA-histone complexes (OR 1.64, 95% CI 1.10–2.44, *P* = 0.016) and MPO-DNA complexes (OR 1.94, 95% CI 1.23–3.06, *P* = 0.001) were at higher risk of developing PPCs at 7 days post-surgery (Table 2). In contrast, other parameters, such as age, sex, weight and transfusion volume, did not show any correlation with the development of PPCs.

**Table 2:** Logistic Regression for PPCs

	**Univariate Analysis**		**Multivariate Analysis**	
**Characteristics**	**OR(95% CI)**	***P***	**OR(95% CI)**	***P***
Age	1.13(0.88–1.25)	0.305	1.69(0.76–1.98)	0.403
Male (vs female)	1.27(0.73–2.59)	0.427	1.82(0.62–2.23)	0.667
Weight	1.38(0.55–2.37)	0.593	0.82(0.78–1.98)	0.532
Transfusion volume	1.19(0.65–3.31)	0.681	0.78(0.43–2.01)	0.786
Massive ascites	1.41(0.82–2.92)	0.775	0.94(0.76–3.33)	0.842
Liquid discharge volume	1.78(0.72–2.45)	0.868	1.56(0.69–2.83)	0.464
Postoperative pain control	1.22(0.34–2.16)	0.930	0.85(0.48–1.63)	0.126
Inhaled oxygen concentration	1.04(0.92–1.35)	1.035	1.21(0.82–1.57)	1.136
cfDNA	2.36(1.82–4.68)	0.000	2.24(1.37–3.65)	0.004
Extracellular DNA-histone complex	1.65(1.18–2.30)	0.003	1.64(1.10–2.44)	0.016
MPO-DNA complex	2.91(1.58–3.52)	0.004	1.94(1.23–3.06)	0.001

*cfDNA* Circulating free DNA

*MPO* Myeloperoxidase

*PPCs* Postoperative pulmonary complications

### Predictive value of post-PLT NET component levels on the occurrence of PPCs

**Fig. 3:**
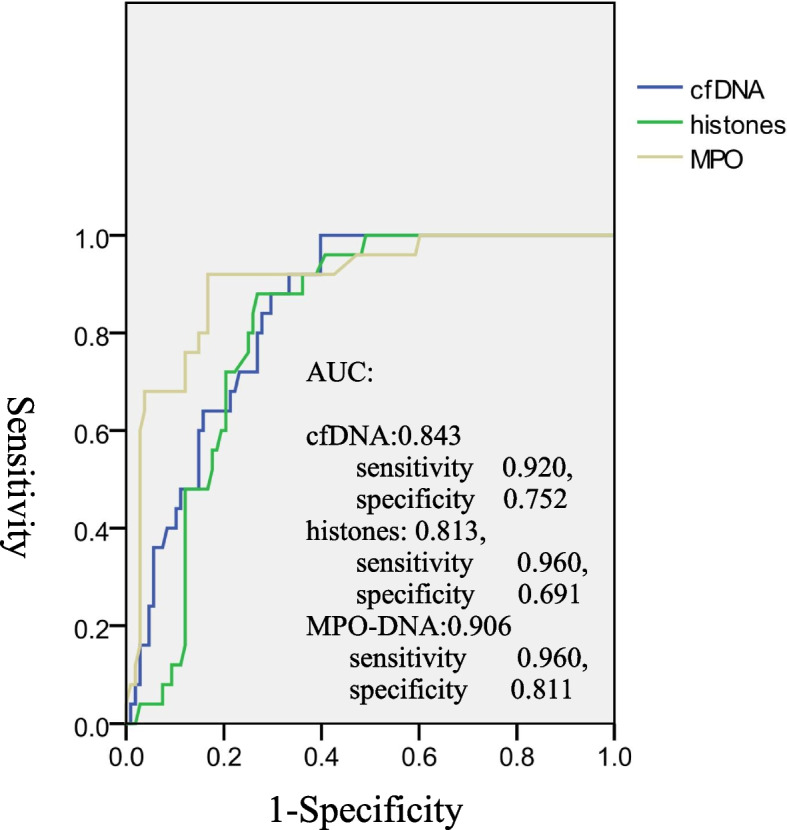
Receiver operating characteristic curve analysis of NET components on PPCs. Receiver operating characteristic (ROC) curve analysis of post-PLT serum NET components with the occurrence of PPCs. The sensitivity, specificity, and area under the curve (AUC) were analysed for cfDNA, DNA-histone complexes and MPO-DNA complexes

To assess the predictive value of NET components on the occurrence of PPCs, we conducted a receiver operating characteristic (ROC) curve analysis. The areas under the curve (AUCs) of cfDNA, DNA-histone complexes, and MPO-DNA complexes for the prediction of PPCs were 0.843 (95% CI 0.776–0.911, sensitivity 0.920, specificity 0.752), 0.813 (95% CI 0.741–0.885, sensitivity 0.960, specificity 0.691), and 0.906 (95% CI 0.842–0.969, sensitivity 0.960, specificity 0.811), respectively (Fig. 3). These data suggest that all three components can serve as biomarkers for PPC development, among which the serum MPO-DNA complex level has the best performance.

### Regression analysis of post-PLT NET component levels with the secondary endpoints: prolonged ICU stay and death within 1 year

We then evaluated the association between NET components and prolonged ICU stay. Logistic regression analysis showed that increased serum levels of cfDNA, DNA-histone complexes and MPO-DNA complexes were associated with prolonged ICU length of stay (cfDNA, OR 1.98, 95% CI 1.16–2.76, *P* = 0.03; DNA-histone complex, OR 4.26, 95% CI 2.11–6.49, *P* < 0.001; MPO-DNA complex, OR 3.69, 95% CI 2.51–5.82, *P* = 0.01) and death within 1 year (cfDNA, OR 1.53, 95% CI 1.38–2.66, *P* = 0.04; DNA-histone complex, OR 2.65, 95% CI 1.78–3.99, *P* = 0.03; MPO-DNA complex, OR 1.85, 95% CI 1.09–4.27, *P* = 0.04) (Table 3). These data suggest that NET components are also acceptable biomarkers to predict the risk of prolonged ICU stay and death within 1 year.

**Table 3:** Logistic regression analysis of NET components with secondary endpoints

	cfDNA		Extracellular DNA-histone complex		MPO-DNA complex	
Clinical Outcomes	OR(95% CI)	*P*	OR(95% CI)	*P*	OR(95% CI)	*P*
Prolonged ICU stay	1.98(1.16–2.76)	0.03	4.26(2.11–6.49)	< 0.001	3.69(2.51–5.82)	0.01
Death within 1 year	1.53(1.38–2.66)	0.04	2.65(1.78–3.99)	0.03	1.85(1.09–4.27)	0.04

*cfDNA* Circulating free DNA

*ICU* Intensive care unit

*MPO* Myeloperoxidase

*NETs* Neutrophil extracellular traps

### PMA-induced NET formation

**Fig. 4:**
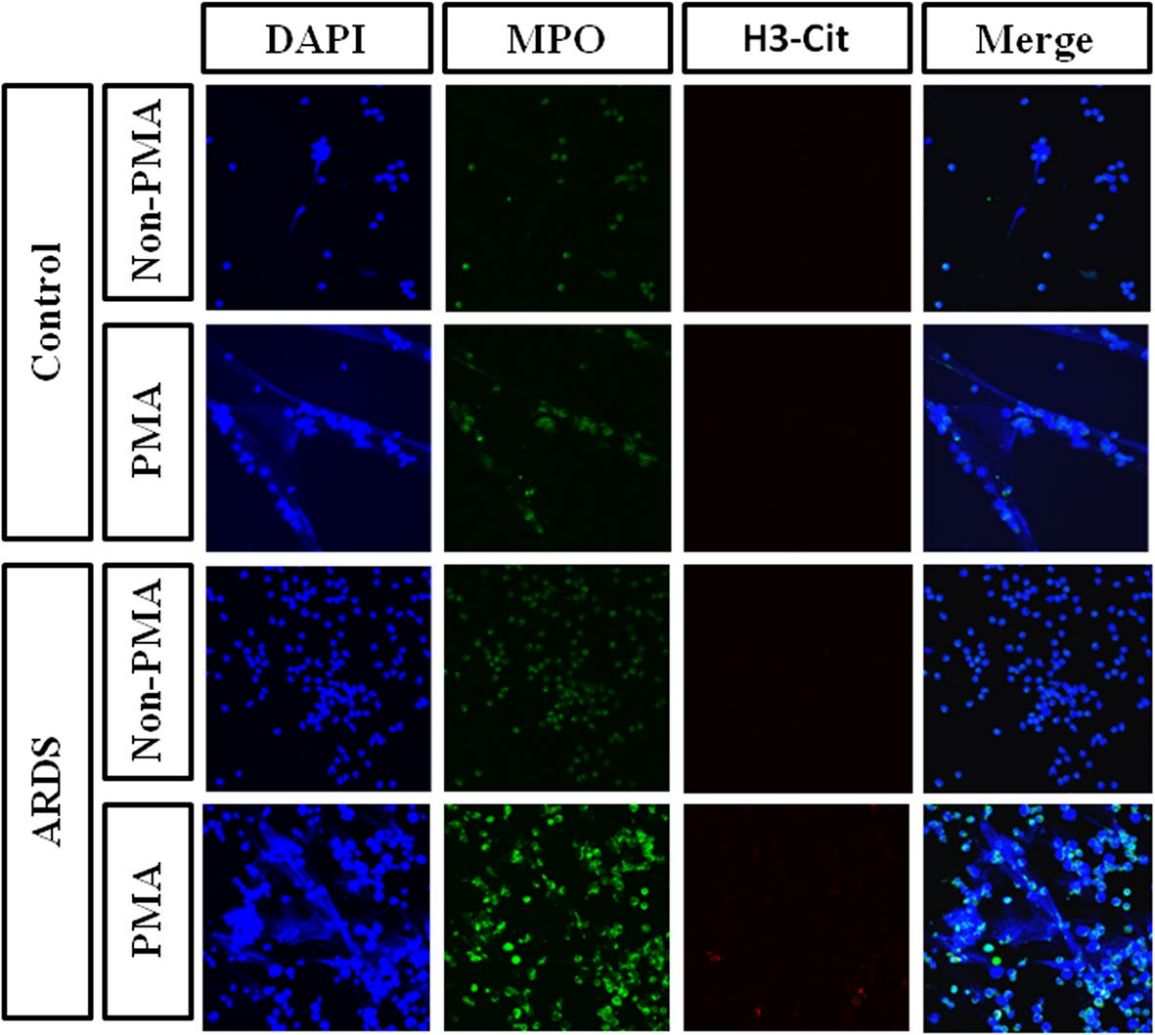
PMA-induced NET formation in vitro. cfDNA, MPO and citrullinated histone 3 were significantly formed in the serum of ALI/ARDS patients compared to the control. After PMA stimulation, these components of NETs increased significantly

We further explored whether PMA could induce NET formation in vitro in both control and ALI/ARDS patients via confocal microscopy. As shown in Fig. 4, cfDNA, MPO and citrullinated histone 3 were significantly elevated in ALI/ARDS patients compared to controls. After PMA stimulation, these components of NETs increased significantly.

### Survival analysis

**Fig. 5:**
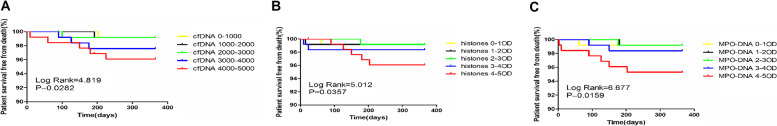
Survival analysis within one year post-PLT. Kaplan‒Meier survival curves during the one-year follow-up based on stratified serum levels of NET components: (**A**) cfDNA, (**B**) DNA-histone complex, and (**C**) MPO-DNA complex

During the one-year follow-up after PLT, 9 of 63 (14.3%) patients died in the PPC group, whereas only 2 of 70 (2.9%) died in the non-PPC group. Kaplan‒Meier survival analysis was performed based on the stratification of NET components after categorical transformation. As shown in Fig. 5, with the increase in serum levels of cfDNA, DNA-histone complexes and MPO-DNA complexes, the survival decreased (the log ranks were 4.819, 5.012 and 6.677, with P values of 0.0282, 0.0357 and 0.0182, respectively). These data suggest that serum NET components may also serve as prognostic markers.

## Discussion

In the present study, we prospectively recruited paediatric patients undergoing PLT and analysed the association between serum concentrations of NET components and the incidence of PPCs, as well as the occurrence of prolonged ICU stay and the death rate within 1 year. Baseline characteristics and demographic data were balanced between PPC and non-PCC patients, suggesting no selection bias in our study. We found that increased serum levels of cfDNA, DNA-histone complexes and MPO-DNA complexes were associated with a higher risk of PPCs after surgery. In addition, increased NET components were also associated with prolonged ICU length of stay and death within one year. Serum NET components could serve as biomarkers for the development of post-PLT PPCs and to evaluate prognosis.

Liver transplantation is a successful treatment for patients with acute liver failure and end-stage liver cirrhosis. The morbidity and mortality associated with liver transplantation continue to decrease due to refinements in surgical technique, immunosuppression, and imaging [25]. However, despite advances in medical and surgical treatment, PPCs continue to be a major source of morbidity and mortality, with an incidence ranging from 42.1% to 96.5% [26]. The most frequent types of PPCs in our study were identical to those in the published literature: pleural effusion, pulmonary oedema, atelectasis, pneumonia and ARDS. PPCs, especially ARDS, contribute to the unfavourable prognosis of liver transplant recipients [27]. However, the mechanisms and prediction methods are still under investigation. In this regard, our study is an important complement to the field, suggesting possible pathobiological effects of NETs in PPCs and the potential value of serum NET components as biomarkers.

Since its first discovery in 2004 [28], NETs have drawn increasing attention in host defence, autoimmunity, and inflammatory disorders across multiple organs. Two types of NETs have been identified: suicidal NETs and vital NETs [29]. Suicidal NETs involve the generation of ROS and subsequent neutrophil death, whereas vital NETs are oxidant-independent and retain neutrophil functions. Which type of NET is more predominant in post-PLT PPCs remains unclear and was not distinguished in the present study. Future studies focusing on this aspect may reveal the underlying mechanisms of post-PLT PPCs.

Liver transplantation is associated with substantial cell death and intrahepatic neutrophil accumulation [30]. The lung is a vulnerable organ to neutrophil-mediated injuries. Marginating neutrophils residing within the pulmonary vasculature could directly damage the blood-gas barrier upon activation. Liver ischaemia–reperfusion injury in PLT could trigger the release of inflammatory cytokines, the translocation of endotoxin to the systemic circulation, and systemic oxidative stress, all of which could activate neutrophils, leading to lung injury [7]. In addition, activated neutrophils also play a role in coagulation, leading to microthrombus formation and even disseminated intravascular coagulation. These factors could further disrupt the pulmonary vasculature and exacerbate lung injury [15].

NET components may also directly contribute to lung injuries. Histones are known to possess cytotoxic properties against both microorganisms [31] and eukaryocytes [32]. Extracellular histones behave as major mediators of cell damage and organ dysfunction during the hyperinflammatory reaction [33]. A neutralizing antibody against histone H3 reduced mortality in an experimental model of murine sepsis [34]. In addition, histones activate a wide spectrum of platelet responses, such as platelet aggregation and thrombus formation [35]. Recent studies have shown that NETs induce platelet adhesion and aggregation [36, 37]. NETs also provide a previously unrecognized scaffold and facilitate thrombus formation [35]. Indeed, treatment with the NET inhibitor peptidyl-arginine-deiminase 4 significantly protected hepatocytes from injury by inhibiting coagulation after liver transplantation [38]. MPO is a neutrophil granule enzyme that is a part of the oxygen-dependent antimicrobial defence system and catalyses hypochlorous acid formation [39]. In neutrophils, MPO is carefully retained in organelles such as phagolysosomes. Free floating MPO causes direct tissue damage and inflammation [40].

In the present study, we proved that serum NET components correlate well with the occurrence of post-PLT PPCs. It is worth noting that neutrophil activation and NET formation can be both the cause and the consequence of PPCs. One should be cautious when interpreting our data. We did not show direct evidence that NETs result in post-PLT PPCs, although this is biologically plausible. Instead, a high level of NET components could be regarded as a biomarker to warn physicians of the probable development of PPCs. We also report an increased death rate in patients with high levels of NET components, which is consistent with reports for lung injuries from other aetiologies [41] such as COVID-19. It remains to be explored whether high levels of NET components suggest the presence of more “primed” neutrophils or whether the fatality is purely derived from sequelae of the PPCs.

It is worth noting that, apart from NETs, lysis of dead cells may also be a source of cfDNA. For example, tumour-derived cfDNA is particularly high in cancer patients, especially those with advanced disease [42]. The method used to detect cfDNA in the present study cannot identify the source of the cfDNA. A combination of multiple NET components, as in the present study, is preferred to increase the specificity.

Another limitation of the present study is the relatively small sample size. We only included 133 paediatric patients. However, for the primary and secondary outcomes evaluated in our study, the sample size is acceptable. Given the information provided by our study, a power analysis can be performed to optimize the sample size for future studies.

## Conclusions

In our prospective, observational cohort study in paediatric patients undergoing PLT, serum levels of NET components could serve as a biomarker for the development of PPCs. High levels of NET components are also a risk factor for prolonged ICU stay. In addition, these NET components could be used as a prognostic indicator of death within one year.

## Data Availability

The datasets used during the current study are available from the corresponding author on reasonable request.

## References

[1] Cuenca AG, Kim HB, Vakili K (2017). Pediatric liver transplantation. Semin Pediatr Surg.

[2] Gadre S, Kotloff RM (2017). Noninfectious pulmonary complications of liver, heart, and kidney transplantation: an update. Clin Chest Med.

[3] Cardoso FS, Karvellas CJ (2019). Respiratory complications before and after liver transplant. J Intensive Care Med.

[4] Bozbas SS, Eyuboglu FO, Ozturk Ergur F, Gullu Arslan N, Sevmis S, Karakayali H, Haberal M (2008). Pulmonary complications and mortality after liver transplant. Exp Clin Transplant.

[5] Barjaktarevic I, Cortes Lopez R, Steadman R, Wray C, Qadir N, Chang SY, Wang T (2018). Perioperative considerations in liver transplantation. Semin Respir Crit Care Med.

[6] Mack CL, Millis JM, Whitington PF, Alonso EM (2000). Pulmonary complications following liver transplantation in pediatric patients. Pediatr Transplant.

[7] Lui JK, Spaho L, Holzwanger E, Bui R, Daly JS, Bozorgzadeh A, Kopec SE (2018). Intensive care of pulmonary complications following liver transplantation. J Intensive Care Med.

[8] Abrams ST, Zhang N, Manson J, Liu T, Dart C, Baluwa F, Wang SS, Brohi K, Kipar A, Yu W (2013). Circulating histones are mediators of trauma-associated lung injury. Am J Respir Crit Care Med.

[9] Mikacenic C, Moore R, Dmyterko V, West TE, Altemeier WA, Liles WC, Lood C (2018). Neutrophil extracellular traps (NETs) are increased in the alveolar spaces of patients with ventilator-associated pneumonia. Crit Care.

[10] Wang N, Yang X, Sun J, Sun Z, Ma Q, Wang Z, Chen Z, Wang Z, Hu F, Wang H (2019). Neutrophil extracellular traps induced by VP1 contribute to pulmonary edema during EV71 infection. Cell Death Discov.

[11] Karki P, Birukov KG, Birukova AA (2020). Extracellular histones in lung dysfunction: a new biomarker and therapeutic target?. Pulm Circ.

[12] Papayannopoulos V (2018). Neutrophil extracellular traps in immunity and disease. Nat Rev Immunol.

[13] Lin WC, Fessler MB. Regulatory mechanisms of neutrophil migration from the circulation to the airspace. Cell Mol Life Sci. 2021;78(9):4095–124. 10.1007/s00018-021-03768-z PMC7863617 33544156

[14] Frantzeskaki F, Armaganidis A, Orfanos SE (2017). Immunothrombosis in acute respiratory distress syndrome: cross talks between inflammation and coagulation. Respiration.

[15] Evans CE, Zhao YY (2017). Impact of thrombosis on pulmonary endothelial injury and repair following sepsis. Am J Physiol Lung Cell Mol Physiol.

[16] Gould TJ, Vu TT, Swystun LL, Dwivedi DJ, Mai SH, Weitz JI, Liaw PC (2014). Neutrophil extracellular traps promote thrombin generation through platelet-dependent and platelet-independent mechanisms. Arterioscler Thromb Vasc Biol.

[17] Laridan E, Martinod K, De Meyer SF (2019). Neutrophil extracellular traps in arterial and venous thrombosis. Semin Thromb Hemost.

[18] Stakos DA, Kambas K, Konstantinidis T, Mitroulis I, Apostolidou E, Arelaki S, Tsironidou V, Giatromanolaki A, Skendros P, Konstantinides S (2015). Expression of functional tissue factor by neutrophil extracellular traps in culprit artery of acute myocardial infarction. Eur Heart J.

[19] Ducroux C, Di Meglio L, Loyau S, Delbosc S, Boisseau W, Deschildre C, Ben Maacha M, Blanc R, Redjem H, Ciccio G (2018). Thrombus neutrophil extracellular traps content impair tPA-induced thrombolysis in acute ischemic stroke. Stroke.

[20] Maruchi Y, Tsuda M, Mori H, Takenaka N, Gocho T, Huq MA, Takeyama N. Plasma myeloperoxidase-conjugated DNA level predicts outcomes and organ dysfunction in patients with septic shock. Crit Care. 2018;22(1):1–10. 10.1186/s13054-018-2109-7 PMC6045839 30005596

[21] Canet J, Gallart L, Gomar C, Paluzie G, Vallès J, Castillo J, Sabaté S, Mazo V, Briones Z, Sanchis J (2010). Prediction of postoperative pulmonary complications in a population-based surgical cohort. Anesthesiology.

[22] Cao B, Huang Y, She DY, Cheng QJ, Fan H, Tian XL, Xu JF, Zhang J, Chen Y, Shen N (2018). Diagnosis and treatment of community-acquired pneumonia in adults: 2016 clinical practice guidelines by the Chinese thoracic society. Chin Med Assoc Clin Respir J.

[23] Ranieri VM, Rubenfeld GD, Thompson BT, Ferguson ND, Caldwell E, Fan E, Camporota L, Slutsky AS (2012). Acute respiratory distress syndrome: the Berlin Definition. JAMA.

[24] Niewińsk G, Raszeja-Wyszomirska J, Główczyńska R, Figiel W, Zając K, Kornasiewicz O, Zieniewicz K, Grąt M (2018). Risk factors of prolonged icu stay in liver transplant recipients in a single-center experience. Transplant Proc.

[25] Craig EV, Heller MT (2021). Complications of liver transplant. Abdom Radiol (NY).

[26] Aydin C, Otan E, Akbulut S, Karakas S, Kayaalp C, Karagul S, Colak C, Gonultas F, Yilmaz S (2015). Postoperative pulmonary complications after liver transplantation: assessment of risk factors for mortality. Transplant Proc.

[27] Ulubay G, Kirnap M, Er Dedekarginoglu B, Kupeli E, Oner Eyuboglu F, Haberal M (2015). Awareness of respiratory failure can predict early postoperative pulmonary complications in liver transplant recipients. Exp Clin Transplant.

[28] Brinkmann V, Reichard U, Goosmann C, Fauler B, Uhlemann Y, Weiss DS, Weinrauch Y, Zychlinsky A (2004). Neutrophil extracellular traps kill bacteria. Science.

[29] Tan C, Aziz M, Wang P: The vitals of NETs. J Leukoc Biol 2020. 10.1002/JLB.3RU0620-375R PMC9059135 33378572

[30] von Meijenfeldt FA, Burlage LC, Bos S, Adelmeijer J, Porte RJ, Lisman T (2018). Elevated plasma levels of cell-free dna during liver transplantation are associated with activation of coagulation. Liver Transpl.

[31] Kawasaki H, Iwamuro S (2008). Potential roles of histones in host defense as antimicrobial agents. Infect Disord Drug Targets.

[32] Kleine TJ, Gladfelter A, Lewis PN, Lewis SA (1995). Histone-induced damage of a mammalian epithelium: the conductive effect. Am J Physiol.

[33] Xu J, Zhang X, Pelayo R, Monestier M, Ammollo CT, Semeraro F, Taylor FB, Esmon NL, Lupu F, Esmon CT (2009). Extracellular histones are major mediators of death in sepsis. Nat Med.

[34] Semple JW, Freedman J (2010). Platelets and innate immunity. Cell Mol Life Sci.

[35] Fuchs TA, Brill A, Duerschmied D, Schatzberg D, Monestier M, Myers DD, Wrobleski SK, Wakefield TW, Hartwig JH, Wagner DD (2010). Extracellular DNA traps promote thrombosis. Proc Natl Acad Sci U S A.

[36] Carestia A, Kaufman T, Schattner M (2016). Platelets: new bricks in the building of neutrophil extracellular traps. Front Immunol.

[37] Semeraro F, Ammollo CT, Morrissey JH, Dale GL, Friese P, Esmon NL, Esmon CT (2011). Extracellular histones promote thrombin generation through platelet-dependent mechanisms: involvement of platelet TLR2 and TLR4. Blood.

[38] Huang H, Tohme S, Al-Khafaji AB, Tai S, Loughran P, Chen L, Wang S, Kim J, Billiar T, Wang Y (2015). Damage-associated molecular pattern-activated neutrophil extracellular trap exacerbates sterile inflammatory liver injury. Hepatology.

[39] Parker H, Albrett AM, Kettle AJ, Winterbourn CC (2012). Myeloperoxidase associated with neutrophil extracellular traps is active and mediates bacterial killing in the presence of hydrogen peroxide. J Leukoc Biol.

[40] Aratani Y (2018). Myeloperoxidase: Its role for host defense, inflammation, and neutrophil function. Arch Biochem Biophys.

[41] Yang SC, Tsai YF, Pan YL, Hwang TL. Understanding the role of neutrophils in acute respiratory distress syndrome. Biomed J. 2021;44(4):439-46. 10.1016/j.bj.2020.09.001 PMC7481802 33087299

[42] Shaw JA, Stebbing J (2014). Circulating free DNA in the management of breast cancer. Ann Transl Med.

